# Review of Ethnobotanical, Phytochemical, and Pharmacological Study of *Thymus serpyllum* L.

**DOI:** 10.1155/2015/101978

**Published:** 2015-07-22

**Authors:** Snežana Jarić, Miroslava Mitrović, Pavle Pavlović

**Affiliations:** Department of Ecology, Institute for Biological Research “Siniša Stanković”, University of Belgrade, Bulevar Despota Stefana 142, 11060 Belgrade, Serbia

## Abstract

*Thymus serpyllum* L. (wild thyme) is a perennial shrub, native to areas of northern and central Europe. Its aerial parts are most frequently used in ethnomedicine (mainly for treating illnesses and problems related to the respiratory and gastrointestinal systems), although recently its essential oils are becoming more popular as an important plant-derived product. The composition of these oils is affected by geographic region, the development stage of the plant, the harvest season, habitat, and climatic conditions. Wild thyme essential oil has an ever-growing number of uses in contemporary medicine due to its pharmacological properties: antioxidative, antimicrobial, and anticancerogenic activities. The antioxidative and antimicrobial properties of the essential oil are related to the synergistic and cumulative effect of its components. In terms of antitumor and cytotoxic activity, further research into the effects of essential oil is necessary, aimed at improving its cytotoxic effects, on the basis of which appropriate medicines can be formulated. Due to its pharmacological properties, the essential oil of wild thyme, a plant used in traditional medicine, represents an important natural resource for the pharmaceutical industry. In addition, it can be a source of natural antioxidants, nutritional supplements, or components of functional foods in the food industry.

## 1. Introduction

*Thymus serpyllum* L. (wild thyme) belongs to the family Lamiaceae, which according to the World Checklist contains 7534 species [1], including the genus* Thymus* L. with 220 species [2]. This genus is very complex from the taxonomical and systematic points of view, demonstrating significant polymorphism not only in morphological characteristics but also in composition of ethereal oils.

**Figure 1:**
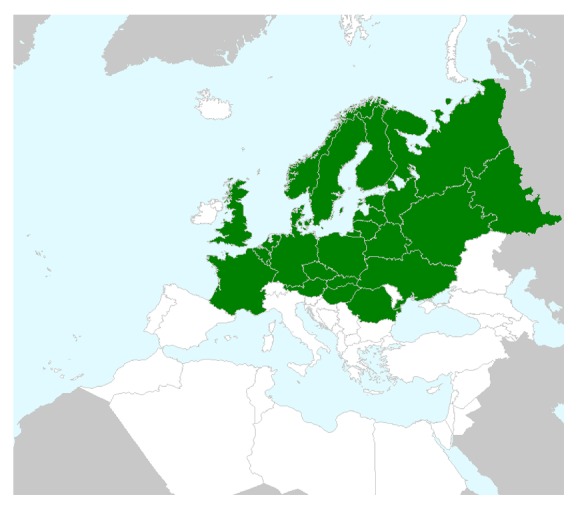
Map of distribution,* Thymus serpyllum* L. (Source: Botanical Museum, Helsinki, Finland, 2014, data from BGBM, Berlin-Dahlem, Germany.)

**Figure 2:**
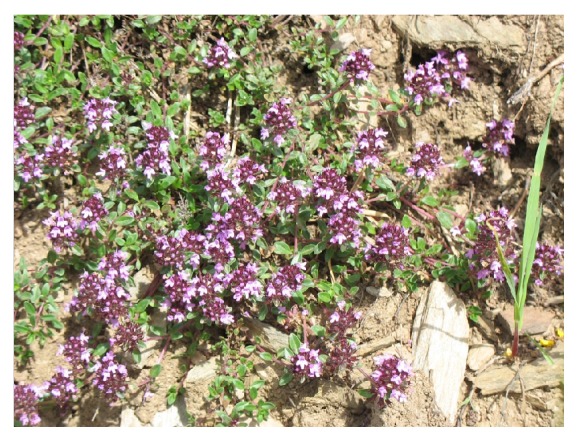
*Thymus serpyllum* L.

*T. serpyllum* L. is a perennial shrub, native to regions of northern and central Europe (Figure 1). It is known as Breckland thyme, wild thyme, or creeping thyme; however, its specific name “*serpyllum*” is derived from the Greek word meaning “to creep,” because of wild thyme's trailing habit. It has a long stem, which is woody at the base but with a sterile leaf rosette at the top. Leaves are oval (rounded at the top, tapered at the base), 4–6 mm long, 2–4 mm wide, and glabrous on the face and underside, while at the base along the edge they have long trichomes, a prominent central vein, and less prominent lateral veins (Figure 2). Inflorescences are 4–7 cm tall and form in a series along a low-lying stem, with a uniform layer of trichomes on all sides. Flowers are located at the top of the stems and form spherical (or more rarely elongated) verticillaster [3]. It flowers from May to September. Wild thyme grows best on dry, stony ground, open sandy heaths, and grasslands.

The medicinal properties of wild thyme have been extensively used in official and traditional medicine for many years and centuries, respectively. Fresh and dried herbs particularly the upper part of the above ground portion of wild thyme, collected when the plant is in bloom, possess certain healing properties due to the presence of significant amounts of essential oils. Recent years have seen increased interest in ethnobotanical, phytochemical, and pharmacological investigations into the medicinal properties of the species* T. serpyllum* which serves as a high quality source for many different formulations in pharmaceutical and chemical industries. The herb is used in preparations of natural herbal remedies, such as syrups, tinctures, infusions, decoctions, tea, and oil. The increase in multidrug resistant strains of pathogenic microorganisms has led to extensive phytochemical and pharmacological studies of* T. serpyllum* as an important source of medicinal substances with antioxidant, antimicrobial, antitumor, and cytotoxic properties and their effective medicinal application, as well as use in pharmaceutical, food, and cosmetic industries. In addition, the increased pressure from consumers for natural products as supplements and their clinical application instead of synthetic chemicals, which are generally perceived by the public as being more toxic, has also stimulated research into many medicinal and aromatic plants of which* T. serpyllum* occupies a very important place.

## 2. Traditional Uses and Ethnopharmacology

The widespread use of different species of the* Thymus* genus dates back to ancient Egypt, where they were used for making perfumed balms, for embalming, and for medical purposes. The Greeks and Romans used them in the same way, as we know from the writings of Pliny (1st century), Dioscorides (2nd century), and Philippus Aureolus Theophrastus Bombastus von Hohenheim (Paracelsus 1493/1494–1541). “Everyone knows thyme,” wrote physician Dioscorides in the first line of his discourse on the pharmacological value of this very aromatic herb, a subject supported by more than three millennia of experience. According to Dioscorides, thyme was used to treat asthma and loosen congestion in the throat and stomach [4]. In terms of geography, the use of these plants spread no further north than the Alps. The first recorded information on the medicinal properties of thyme north of the Alps can be found in the manuscript* Physica*, by the abbess Hildegard von Bingen (1098–1179) and the works of Albertus Magnus (1193–1280). This continued in the 16th century with the Herbal by the herbalist P. Mathiolus (1505–1577), which first mentions the strength and efficacy of thyme. Since then, numerous therapeutic properties have been attributed to thyme, some on an empirical basis, others more debatable [5]. However, the spread of thyme throughout Europe is thought to be due to the Romans, as they used it to purify their rooms and to “give an aromatic flavour to cheese and liqueurs” [6]. In the European Middle Ages, the herb was placed beneath pillows to aid sleep and ward off nightmares [7]. In this period, women would often also give knights and warriors gifts that included thyme leaves, as it was believed to bring courage to the bearer. The pharmacological manuscripts of the Chilandar Medical Codex (15th-16th centuries) mention the use of wild thyme for the treatment of headaches caused by colds, laryngitis, and diseases of the digestive organs and as an antitussive [8]. During the Renaissance period (16th and 17th centuries), wild thyme was used internally to treat malaria and epilepsy [9].

The aerial part of* T. serpyllum* has a long tradition of being used in many countries of Europe [10] and worldwide as an anthelmintic, a strong antiseptic, an antispasmodic, a carminative, deodorant, diaphoretic, disinfectant, expectorant, sedative, and tonic [11]. It is most frequently used for treating illnesses and problems related to the gastrointestinal and respiratory systems [12–19]. In the Western Balkans, this species has an important use as a sedative [16, 20], or to improve blood circulation, and then as anticholesterolemic and immunostimulant [21]. In alpine region of northeastern Italy, infusion or decoction of plant areal parts (in flowering stage) is used in treatment of rheumatism [22]. Gairola et al. mention the use of wild thyme in some regions of India for treating menstrual disorders [23], while Shinwari and Gilani state its use as an anthelmintic in Northern Pakistan [24].* T. serpyllum* is also used externally as an antiseptic, to treat wounds [14], to combat eczema [13], or to reduce swelling [25]. In some areas of Italy, wild thyme is used as an important herb in cookery, mainly for flavouring meat or fish [26]. In addition, ethnobotanical studies in Catalonia and Balearic Islands have proved usage of* T. serpyllum* in ethnoveterinary particularly as antidiarrheal [27]. The* British Herbal Pharmacopoeia* classifies this species as a medicinal plant and among the indications for its use it mentions bronchitis, bronchial catarrh, whooping cough, and sore throats. Whooping cough is singled out as a specific indication. In the monograph, recommendations are given for combining it with other plants (Coltsfoot,* Tussilago farfara* L., or Horehound,* Marrubium vulgare* L.). As a gargle for acute pharyngitis, it is recommended in combination with the leaves of blackberry (*Rubus fruticosus* L.) or* Echinacea* (*Echinacea* sp.) [28]. According to the PDR for Herbal Medicines, wild thyme is a component in various standardized preparations with antitussive effects, while alcohol extracts are integral components of drops used for coughs and colds [29]. The recommended daily dose of this drug is 4–6 g.

## 3. Pharmacological Properties

Many studies on the chemical composition and yields of the essential oils from plants belonging to the* Thymus* genus have been conducted, including those from* T. serpyllum*. The chemical composition and yield of the essential oil of* T. serpyllum* are considered to be affected by geographic region, the development stage of the plant, the harvest season, habitat, and climatic conditions [35]. As such, its content varies from 0.1 to 0.6% [29, 36, 37] or even from 0.1 to 1% [38]. Analysis of the yield of essential oil of* T. serpyllum* in Estonia revealed its content to be between 0.6 and 4.4 mL/kg. Only in one locality did it amount to 3 mL/kg [39], which is in accordance with European Pharmacopoeia standards. Similarly, the essential oil content of wild thyme from 5 regions in Armenia ranged between 4.5 and 7.4 mL/kg [40]. In samples of wild thyme from Pakistan, yields of 0.48% were achieved [41], or 29 g/kg [42]. In Serbia, the yields of essential oil from samples of this species growing on Mt. Kopaonik were 3 mL/kg (~0.3%) [43] and 4.1 g/kg (~0.1%) in samples from Mt. Pasjača [44].

Over the last two decades, more and more studies have researched the chemical composition of* T. serpyllum* essential oil (Table 1) [39, 41–50]. It has been established that plant species of the* Thymus* genus are characterized by chemical polymorphism, meaning that several chemotypes exist (geraniol, germacrene D, citral, linalool, (E)-caryophyllene, *α*-terpinyl acetate, carvacrol, and thymol) [30, 33, 51]. According to the PDR for Herbal Medicines, the chief component of the essential oil of* T. serpyllum* is carvacrol, while it also contains borneol, isobutyl acetate, caryophyllene, 1,8-cineole, citral, citronellal, citronellol,* p*-cymene, geraniol, linalool, *α*-pinene, *γ*-terpinene, *α*-terpineol, terpinyl acetate, and thymol in relatively high concentrations [29]. Carvacrol and thymol are isomers, belonging to the group of monoterpenic phenols with powerful antiseptic properties. They are very quickly absorbed after application and quickly metabolise as they are not subject to first phase biotransformation; instead their conjugation with sulphuric and glucuronic acids occurs directly. They are excreted via urine within 24 hours, mainly in the form of conjugates, and less so in their unchanged form [32]. According to the European Pharmacopeia, the herb* T. serpyllum* must contain at least 1.2% essential oil, in which the total content of carvacrol and thymol is 40% or higher [31]. In addition to essential oil, wild thyme also contains flavonoids, phenol carboxylic acids, and their derivatives, triterpenes and tannins [29, 52]. Besides carvacrol and thymol, Kulišić et al. also include *γ*-terpinene and p-cymene among the main components of the essential oil [53]. However, research into the composition and concentration of compounds in the essential oil of* T. serpyllum* in different regions of the world has revealed significant differences. For example, the content of essential oil in populations of wild thyme in the Altai Mountains (Russia) is 0.5–1%, but its chemical composition differs significantly depending on the altitude. In the village of Kolyvan (150 m a.s.l.), the principal components of the oil are *β*-myrcene (4.0%),* p*-cymol (3.8%), 1,8-cineole (14.0%),* cis*-*β*-terpineol (8.2%), camphor (4.0%), and* trans*-nerolidol (29.8%), while in the same region, but in the village of Mendur-Sokkon (500–750 m a.s.l.), the following were identified as the main components:* p*-cymol (14.5%), 1,8-cineole (5.6%), *γ*-terpinene (17.2%), and carvacrol (29.6%) [45]. The essential oil from both areas contained less than 2% thymol. Furthermore, in the essential oil of* T. serpyllum* growing wild in Lithuania, the presence of thymol and carvacrol was not established [54]. Although noted as dominant components in literature, thymol and carvacrol are not the principal components of wild thyme essential oil in Estonia either [40]. Differences in the chemical composition of essential oils have been established in other localities, too: the principal components of the essential oils of wild thyme from Mt. Kopaonik (Serbia) are* trans*-caryophyllene (27.7%), *γ*-muurolene (10.5%), and *α*-humulene (7.5%) [33], while on Mt. Pasjača (Serbia) the dominant components of the essential oil are* trans*-nerolidol (24.2%), germacrene D (16.0%), thymol (7.3%), *δ*-cadinene (3.7%), and *β*-bisabolene (3.3%) [32]. The essential oil of* T. serpyllum* growing in Pakistan contains mainly thymol (53.3%) and carvacrol (10.4%) [41], while Hussain et al., also in Pakistan, but from a different area (the Gilgit Valley), found that the chemical composition of the essential oil was dominated by carvacrol (44.4%) and o-cymene (14.0%) [42]. De Lisi et al. studied the composition of the essential oils of various ecotypes from the region of southern Italy and established that there are differences in the composition between biotypes, too: in two biotypes (S_2_ and S_3_), the concentration of geraniol was highest (35% and 22%, resp.), while in biotype S_1_ thymol is predominant (32.6%) [55]. Ložiene et al. carried out research on 26 samples from 14 habitats in Lithuania and found that there were wide variations in the composition of the main components of the oils [56]. They recorded the existence of five chemotypes (1,8-cineole, germacrene B, (E)-*β*-ocimene, *α*-cadinol, and* cis*-p-ment-2-en-1-ol), which are directly connected to the oil composition among the studied varieties and chemotypes.

Due to the great variability in the content of the same components in* T. serpyllum*, the composition of the essential oil cannot be used as a reliable chemotaxonomic marker. However, its composition is of great importance when it comes to medical and cosmetic uses, as well as in industries where essential oils are used as raw materials. The pleasant fragrance of wild thyme essential oil is mainly down to the phenolic monoterpenoids thymol and carvacrol, which inhibit lipid peroxidation and demonstrate powerful antimicrobial properties on various kinds of microorganisms [34]. Numerous compounds in the composition of the essential oil are natural antioxidants that act in metabolic response to the endogenous production of free radicals and other oxidant species. These responses are due to ecological stress or are promoted by toxins produced by pathogenic fungi and bacteria [57].

### 3.1. Antioxidant Activity

The number of published works studying the antioxidant activity of* T. serpyllum* is relatively small, but some have evaluated it and compared it with other species [42, 44, 46]. For example, Hussain et al. noted that* T. serpyllum* essential oils demonstrated better radical scavenging activity (IC50: 34.8 mg/mL) than* Thymus linearis* (Benth. ex Benth) essential oil (IC50: 42.9 mg/mL) [42]. In terms of inhibition of linoleic acid peroxidation,* T. serpyllum* essential oil again exhibited better antioxidant activity than* T. linearis* essential oil (84.2% and 76.0%, resp.). They also established that thymol, the major component in the essential oil of both species, demonstrated better antioxidant activity than the entire oil whereas carvacrol, a major component of* T. serpyllum* essential oil, exhibited weaker antioxidant activity than the oil itself. Petrović et al. studied the antioxidant capacity of wild thyme essential oil in terms of its ability to neutralise DPPH (1,1-diphenyl-2-picryl-hydrazyl) free radicals, that is, the ability of the components of the essential oil to donate hydrogen atoms and transform DPPH into its reduced form DPPH-H [44]. Their results showed that the essential oil exhibited significantly better antioxidant activity when compared to synthetic antioxidants like butylated hydroxyanisole (BHA) and in particular butylated hydroxytoluene (BHT). Research into the antioxidant capacity of the essential oil of* T. serpyllum* growing in Croatia revealed that it demonstrates poorer ability to neutralise DPPH radicals than BHA, BHT, tocopherol, ascorbic acid, and the essential oil of* T. vulgaris* [46]. Hussain et al. also established that the essential oil of* T. serpyllum* growing in Pakistan exhibited less ability to neutralise DPPH radicals than BHT and thymol [42]. Mihailović-Stanojević et al. proved the pronounced antioxidant activity of wild thyme aqueous extracts containing phenols and flavonoids in terms of their high antioxidant capacity and potential antihypertensive effect on spontaneously hypertensive and normotensive rats [58]. They showed that the bolus injection of this extract (100 mg/kg body weight) decreases systolic and diastolic blood pressure and total peripheral resistance in the former, without affecting these parameters in the latter. The predominant phenolic compounds were rosmarinic and caffeic acids.

The antioxidant activity exhibited by the tested essential oils justifies the traditional uses of wild thyme. Hazzit et al. found that antioxidant potential should be attributed to the phenol constituents of the essential oil [59]. The oil's chemoprotective efficacy against oxidative stress-mediated disorders is mainly due to its free radical scavenging and metal chelating properties. However, the antioxidant activity of the essential oil of* T. serpyllum* is not due to the mere presence of certain dominant components but is the result of the synergism of a larger number of components, including some which are present only in small amounts (*trans*-nerolidol, germacrene D, *δ*-cadinene, and *β*-bisabolene) [34].

### 3.2. Antimicrobial Activity

Many scientists ascribe the antimicrobial activity of species from the* Thymus* genus to the high concentration of carvacrol in its essential oil [60–62]. It has biocidal properties, which lead to bacterial membrane perturbations. Moreover, it may cross cell membranes, reaching the interior of the cell and interacting with intracellular sites vital for antibacterial activities [63, 64]. The biological precursor of carvacrol and another significant component of the plant extracts, p-cymene, has very weak antibacterial properties, but it most likely acts in synergy with carvacrol by expanding the membrane, causing it to become destabilized [65].

An antimicrobial assay revealed that ethanol and aqueous extracts of* T. serpyllum* demonstrated inhibitory activity against* Staphylococcus aureus*,* Bacillus subtilis*,* Escherichia coli*, and* Pseudomonas aeruginosa* tested organisms [66]. A comparative analysis by Lević et al. on the effects of the essential oils of oregano (*Origanum majorana* L.), thyme (*Thymus vulgaris* L.), and wild thyme on the growth of the bacterial species* Proteus mirabilis*,* Escherichia coli*,* Salmonella choleraesuis*,* Staphylococcus aureus*, and* Enterococcus faecalis* revealed that oregano essential oil demonstrated the greatest antimicrobial activity, while wild thyme essential oil had the least inhibitory effect on the growth of these microorganisms [67]. It was research by Ahmad et al. that showed that wild thyme essential oil has bactericidal effects, but not bacteriostatic effects on the bacterial species* Escherichia coli*,* Salmonella Typhi*,* Shigella ferarie*,* Bacillus megaterium*,* Bacillus subtilis*,* Lactobacillus acidophilus*,* Micrococcus luteus*,* Staphylococcus albus*,* Staphylococcus aureus*, and* Vibrio cholera* [41]. Sokolić-Mihalak et al. established that the microbial activity of wild thyme essential oil can be attributed to the effects of its phenolic compounds on inhibiting growth and mycotoxin production of the following species:* Aspergillus ochraceus*,* A. carbonarius*, and* A. niger*, which totalled 60% [68]. The inhibitory activity of essential oils depends on the conditions and duration of incubation, so a greater inhibitory effect is achieved thanks to the synergistic and cumulative effects of the other components of the essential oil. Nikolić et al. found a positive correlation between the antimicrobial activity of selected essential oils of* T. serpyllum*,* Thymus algeriensis*, and* T. vulgaris* and their chemical composition, which indicates that the activity may be ascribed to the phenolic compound thymol because it occurs in high proportions in these oils [50]. This confirms earlier findings that thymol is a good antimicrobial agent [69, 70]. However, although* T. serpyllum* essential oil had the lowest thymol content, it demonstrated the greatest antimicrobial activity, which confirms the importance of the synergistic effect of the other components.

### 3.3. Antitumor and Cytotoxic Activity

As one of the principal constituents of thyme essential oil, carvacrol has important in vitro cytotoxic effects on tumour cells [71]. Experiments have confirmed that carvacrol from* Thymus algeriensis* and different wild varieties of Moroccan thyme demonstrates significant cytotoxic activity against leukaemia P388 in mice [72] and Hep-2 [73]. However, according to Tsukamoto et al., thymol, which is also one of the major constituents in the essential oils of* T. serpyllum*,* T. algeriensis*, and* T. vulgaris*, might be involved in stimulating the active proliferation of pulpal fibroblasts [74]. By comparing the antitumor activities of the essential oils of the species mentioned above on the growth of four human tumour cells, Nikolić et al. confirmed that it is the essential oil of* T. serpyllum* that exhibits the greatest antitumor activity [50]. Namely,* T. serpyllum* was the most potent in all the tested cell lines and contains thymol as its major constituent, a phenolic compound known in literature for its antiproliferative activity [75].

Of the 21 compounds isolated, carvacrol, thymol, and thymoquinone are the major components of hexane extract of* Thymus serpyllum* essential oil, and the hexane extract of this species is cytotoxic to 6 cancer cell lines (MDA-MB-231, MCF-7, HepG2, HCT-116, PC3, and A549). It demonstrated the best anticancer activity in HepG2 (Liver Carcinoma Cell Line), followed by HCT-116 (Colon Cancer Cell Line), MCF-7 (Breast Cancer Cell Line), MDA-MB-231 (Breast Cancer Cell Line), PC3 (Prostate Cancer Cell Line), and A549 (Lung Carcinoma Cell Line), as proved by Baig et al. [76].

## 4. Conclusions

*T. serpyllum* has a tradition stretching back many centuries of being used in ethnomedicine as an aromatic, analgesic, antiseptic, diaphoretic, anthelmintic, expectorant, diuretic, spasmolytic, carminative, sedative, stimulant, and tonic. The aerial part of the plant has traditionally been most frequently used in the treatment of illnesses and problems related to the respiratory, digestive, and urogenital tracts. However, the use of the essential oil, as one of the important plant-derived products of this species, is increasing in contemporary medicine due to its pharmacological properties. The chemical composition and yield of the essential oil of* T. serpyllum* are considered to be affected by geographic region, the development stage of the plant, the harvest season, habitat, and climatic conditions. Therefore, its composition is of great importance when it comes to medical and cosmetic uses, as well as in industries where essential oils are used as raw materials. Novel studies have revealed its pronounced antioxidant and antimicrobial properties of the essential oil being based on the synergistic and cumulative effect of its components. At the same time,* T. serpyllum* essential oils demonstrated better overall antioxidant activity in comparison to other* Thymus* species, due to better antioxidant activity of its major component in the essential oil, thymol, than the entire oil and a major component of other* Thymus* species' essential oil, carvacrol. Future research should seek to answer to what extent thymol or carvacrol is responsible individually for cytotoxicity and how much it is the result of the combination with other constituents of the essential oil. In terms of its antitumor and cytotoxic activities, it is our opinion that further research is needed into the effects of the hexane extract, aimed at improving its cytotoxic effects on cancer cell lines, particularly liver cancer, on the basis of which appropriate medicines can be formulated.

Due to its pharmacological characteristics, the essential oil of wild thyme represents an important natural resource for the pharmaceutical industry. Additionally, it is a source of natural antioxidants, nutritional supplements, or components of functional foods in the food industry.

## Figures and Tables

**Table 1:** The chemical composition of the essential oil of *Thymus serpyllum* L. in some regions.

Compound	Regions/locality				
	Estonia [30]	Serbia (Pasjača) [31]	Serbia (Kopaonik) [32]	Pakistan [33]	“Natures” company and local Greek pharmacy in Thessaloniki [34]
Yield percentage					

*Monoterpene hydrocarbons *					
*α*-Pinene	0,649	0,51	6,9	6,06	2,0
Tricyclene	0,062				0,1
Camphene	2,170	0,35	1,0	0,1	2,4
Sabinene	0,187	0,21			0,8
*β*-Pinene	0,374	0,67	1,8	1,43	0,2
*α*-Phellandrene	0,016		0,5	0,05	0,2
Myrcene	6,152	1,64			
*α*-Terpinene	0,041	0,21			1,1
*p*-Cymene	0,410	2,11	2,0		8,9
*o*-Cymene				14,0	
*β*-Cymene				0,19	
Limonene	0,352	1,03	2,7		0,6
*trans*-*β*-Ocimene	0,986	1,55	1,5		0,1
*cis*-*β*-Ocimene	0,069		0,7		
*α*-Thujene	0,047				
*γ*-Terpinene	0,147	1,48	1,4	0,02	7,2

*Oxidized monoterpenes *					
1,8-Cineole	1,247	1,38	2,5	3,44	0,4
*α*-Thujone	0,046				1,1
*cis*-Thujone		1,89			
*trans*-Thujone	0,084	0,21			
*cis*-Sabinene hydrate	0,047				0,5
Linalool	3,000	0,72	1,2	2,02	2,4
*δ* ^3^-Carene					0,1
Terpinolene	0,090				
*α*-Campholen		0,24			
Camphor	3,545	0,99	3,6		0,7
*cis*-Chrysanthenol					0,4
Borneol	4,667	0,56		2,45	6,0
Menthol		0,26			
Isoborneol	0,028				
*p*-Mentha-3,8-diene				0,18	
Terpinene-4-ol	0,594	0,4			0,7
*α*-Terpineol	2,490	0,52		6,47	0,1
*trans*-Sabinene hydrate	0,087				
*cis*-Linalol oxide	0,100				
(Z)-p-Mentha-2,8-dien-1-ol^*∗*^1118	0,017				
(Z)-p-Mentha-2-en-1-ol^*∗*^	0,003				
*cis*-Sabinol^*∗*^	0,206				
*p*-Cymen-8-ol^*∗*^	0,037				
*trans*-Sabinol^*∗*^	0,0017				
(E)-Dihydrocarvone	0,079				
(E)-Carveol	0,088				
Nerol	0,170				
Neral	0,009				
Carvone	0,055				
Thymol methyl ether		0,29			3,8
Fenchyl alcohol				0,14	
Carvacrol methyl ether		0,49			
Thymoquinone		0,43			
Geraniol	0,233	1,42			
Geranial	0,022	0,5			
1-Decanol	0,118				
*cis*-Dihydrocarvone	0,006				0,1
Bornyl acetate	1,291	0,27			7,0
Isobornyl acetate					0,1
*β*-Citronellol				1,15	
Thymol	0,958	7,26	5,6		38,5
Thymol acetate					2,8
Carvacrol	0,620	0,61		44,4	4,7
Carvacrol acetate					0,3
Geranyl acetate	3,303			0,39	
Linalyl acetate	1,170			0,38	
(E)-Sabinyl acetate	0,140				
Terpinyl acetate	0,016	1,01			

*Sesquiterpene hydrocarbons *					
*α*-Copaene	0,191	0,27			
*β*-Copaene		0,42			
*β*-Bourbonene	0,350	0,87			
*α*-Selinene				0,05	
*α*-Ylangene	0,222				
*β*-Longipinene				0,17	
Longifolene				0,56	
*β*-Cubebene		0,64			
*α*-Cubebene				0,29	
*α*-Guaiene^*∗*^	0,021				
Selina-3,7(11)-dien	0,600				
Germacrene B	0,297				
*δ*-Elemene	0,013				
*β*-Elemene	0,076	0,71			
*β*-Caryophyllene	6,222	2,76		5,25	1,3
Longicyclene				0,14	
Amorphene				0,04	
*α*-Humulene	0,300	0,4	7,5	1,11	
Aromadendrene					0,1
Alloaromadendrene	0,285	0,34		0,02	0,1
(E)-*β*-Farnesene	0,092	2,28			
*γ*-Muurolene	0,019	0,54	10,5		
Germacrene D	5,495	16,02			
Germacrene D-4-ol	0,088		1,1		
*epi*-Sesquiphellandrene	0,041	0,22			
Bicyclogermacrene	0,200	0,63			0,2
Valencene				0,04	
*α*-Muurolene	0,237	0,52	1,6	0,05	
*β*-Bisabolene	0,873	3,33	1,9		1,0
*β*-Bisabolol	0,100		2,6		
*trans*-Calamenene		0,97			
Calamenene				0,41	
*δ*-Cadinene	0,655	3,73	1,3		0,2
*α*-Cadinene		0,21			
*γ*-Cadinene	0,320				0,1
*α*-Calacorene		0,21			

*Oxidized sesquiterpenes *					
*cis*-Sesquisabinene hydrate		0,29			
Elemol		0,29			
*trans*-Nerolidol	24,527	24,2	2,4		
Caryophyllene oxide	10,288	1,12	1,3	2,32	0,4
Thujopsene-2-*α*-ol		0,34			
Viridiflorol	0,170	0,61			
*β*-Copaen-4-*α*-ol		0,24			
Humulene-epoxide II	0,106	0,46			
*β*-Oplopenone		0,39			
1,10-Di-epi-cubenol		0,43			
Epi-*α*-cadinol (*τ*-cadinol)		1,47	1,1		0,1
Spathulenol		1,220		0,05	0,3
Cadrol					
Ledol^*∗*^	0,382				
*δ*-Cadinol	0,331				
T-Cadinol	0,773				
T-Muurolol	0,006				
*α*-Farnesol	0,561				
*α*-Santanol	0,197				
*α*-Bisabolol	0,740				
Hedycaryol^*∗*^	0,120				
*α*-Guaiol					0,1
*α*-Muurolol		0,57			
*α*-Cadinol	0,570	2,45	1,6		
Helifolenol A		0,37			
*α*-Eudesmol					0,2
Eudesm-3-en-6-ol					0,6
Germacra-4(15),5,10(14)-trien-1-*α*-ol		1,54			

*Others *					
1-Octen-3-ol	0,724	0,24			0,2
3-Octanol	0,091	0,24			
3-Octanone	0,727		6,6		
2-Heptenol	0,071				
1-Dodecanol	0,152				
Neryl acetate	0,074				
*α*-Ionone	0,040				
n-Heptadecane	0,043				
n-Nonadecane	0,049				
n-Heneicosane	0,079				
